# Challenges in Determining the Location of Dopants, to Study the Influence of Metal Doping on the Photocatalytic Activities of ZnO Nanopowders

**DOI:** 10.3390/nano9030481

**Published:** 2019-03-25

**Authors:** Takuya Tsuzuki, Rongliang He, Aaron Dodd, Martin Saunders

**Affiliations:** 1 Research School of Electric, Energy and Materials Engineering, College of Engineering and Computer Science, Australian National University, Canberra 0200, Australia; 2 Institute for Frontier Materials, Deakin University, Waurn Ponds 3216, Australia; rongliang.he@deakin.edu.au; 3 Centre for Microscopy, Characterization and Analysis, The University of Western Australia, 35 Stirling Highway, Perth 6009, Australia; aaron.dodd@uwa.edu.au (A.D.); martin.saunders@uwa.edu.au (M.S.)

**Keywords:** nanoparticles, photocatalysis, doping, ZnO: transition metal

## Abstract

Impurity doping is one of the common approaches to enhance the photoactivity of semiconductor nanomaterials by increasing photon-capture efficiency in the visible light range. However, many studies on the doping effects have produced inconclusive and conflicting results. There are some misleading assumptions and errors that are frequently made in the data interpretation, which can lead to inconsistent results about the doping effects on photocatalysis. One of them is the determination of the location of dopants. Even using advanced analytical techniques, it is still challenging to distinguish between bulk modification and surface modification. The paper provides a case study of transition-metal-doped ZnO nanoparticles, whereby demonstrating common pitfalls in the interpretation of the results of widely-used analytical methods in detail, and discussing the importance of using a combination of many characterization techniques to correctly determine the location of added impurities, for elucidating the influence of metal doping on the photocatalytic activities of semiconductor nanoparticles.

## 1. Introduction

Zinc oxide (ZnO) is an important semiconducting material, having a broad range of applications including light-emitting devices [1], spintronics [2], transparent conductive oxides [3], and ultraviolet (UV) light blockers [4]. In particular, ZnO is one of the most widely studied photocatalysts [5]. The band gap energy of bulk ZnO is 3.3 eV and thus UV rays can excite valence electrons onto the conduction band. When these photo-excited electrons and holes move to the particle surfaces where water and oxygen molecules reside, highly active free radicals such as superoxide (^•^O_2_^−^) and hydroxyl (^•^OH) are generated and undergo secondary reactions such as the decomposition of organic compounds. Photocatalysis is useful in many applications including clean hydrogen fuel production, organic pollutant scavenging, removal of heavy metals from water, and anti-fouling coatings. Much effort has been made to enhance the photocatalytic property of ZnO [6], including size reduction, facet engineering, photo-sensitization with dyes or quantum-dots, decoration of surface with charge separators, and impurity doping.

Impurity doping of ZnO is normally aimed at creating intra-bandgap energy levels, which allows ZnO to absorb visible-light and utilizes a wider solar spectrum for photocatalysis [7,8]. For this purpose, bulk crystal doping with transition metals, i.e., replacement of zinc ions with transition metal ions to form a solid solution of two semiconductors with identical crystal structures [9], has been widely investigated. For successful bulk modification by crystal doping, the dopant ions should fulfill some requirements. Firstly, the ionic radius of the dopant ions should be similar to that of Zn^2+^ [10]. Ions with a larger ionic radius are difficult to crystal dope and doping of ions with smaller or larger ionic radius will induce high lattice distortion, leading to the formation of physical defects that act as charge recombination sites for photo-generated electrons and holes, leading to decreased photocatalytic activities [7]. Secondly, in the case of doping a single element, ions with a valence number of 2+ are preferred. If the dopant ions do not have the same valence number as zinc ions, the charge imbalance may result in the enhanced recombination of photo-generated electrons and holes so that doping may reduce photocatalytic activity [7].

However, many studies on the doping effects have produced inconclusive and rather conflicting results. This situation greatly hinders progress in developing efficient photocatalysts with desired properties. The reason for the conflicting results is that, as Ohtani discussed thoroughly, there are many key analyses that are frequently missed in these reports [11,12]. One of the most common oversights in the doped semiconductor nanocatalysts is the characterization of doping states, in particular the location of dopant ions [7]. Intended crystal doping (bulk modification) may result in condensation on or complexation of the surface (surface modification) [9], depending on the synthesis conditions. However, the confirmation of bulk doping is often assumed without validation. This is because, contrary to the commonly held view, it is not straightforward to obtain clear evidence for successful bulk crystal doping. Although bulk crystal doping is often assessed by (i) powder X-ray diffraction (XRD) phase analysis, (ii) shift in the position of XRD peaks and (iii) appearance of new optical absorption bands in the absorption spectra, XRD and optical absorbance measurements alone are not sufficient to confirm the successful replacement of zinc ions with dopant ions in ZnO.

This paper aims to demonstrate the challenges in obtaining clear evidence for successful bulk crystal doping, by using ZnO nanoparticles as an example. The paper discusses in detail the common pitfalls in the interpretation of the results of widely-used analytical methods and explains the importance of using a combination of many techniques to correctly determine the location of impurity elements, whereby providing researchers with guidance in the experimental methods and data interpretation for analyzing impurity-added nano-photocatalysts. Here, we focus solely on the determination of the location of impurity ions and detailed discussion of the formation of oxygen defects is put outside the scope of the paper. ZnO is selected as an example material because it is one of the most studied photocatalysts. Co^2+^ and Mn^2+^ are chosen as example impurity elements because they are the most commonly used transition metal elements to modify the photocatalytic activity of ZnO nanoparticles, due to the fact that Shannon’s effective ionic radii for tetrahedrally coordinated Zn^2+^, Co^2+^ and Mn^2+^ are similar to each other, 0.74, 0.72 and 0.80 Å, respectively [13], and the solubility limits of cobalt and manganese in ZnO are higher than other transition metal elements [10]. Nonetheless, the lesson learnt from the examples will be applicable to other doped semiconductor photocatalytic nanoparticles. This paper integrates for the first time the results of some of our previously published papers [14,15,16,17], in order to present a comprehensive explanation of the challenges we face today in interpreting the influence of transition-metal doping on the photocatalytic activities and in distinguishing between bulk modification and surface modification. Additional data analysis was performed and elemental mapping images by high-resolution scanning transmission electron microscopy were newly acquired, which provided vital information in elucidating the appropriate methods to determine the location of impurities/dopants. Details of sample preparation, analytical techniques and the characteristics of resulting particles are given in the supporting document.

## 2. Past Reports on the Effects of Co^2+^ and Mn^2+^ Impurities 

In the past, many attempts were made to modify the photocatalytic activities of ZnO by adding cobalt and manganese impurities. Many techniques have been utilized to synthesize cobalt-doped ZnO, including sol–gel methods and physical sputter deposition methods [18]. Table 1 summarizes some of the recent reports about the effects of cobalt-doping on the photocatalytic activity of ZnO nanoparticles. It can be seen that cobalt-doping generally results in enhanced photocatalytic activity under visible light. This is due to the fact that doped Co^2+^ creates visible-light absorption bands at 659 nm, 615 nm, and 568 nm that are associated with crystal-field d-d* electron transitions of tetrahedrally coordinated Co^2+^ ions; ^4^A_2_**→**^2^E, ^4^A_2_**→**4T1, and ^4^A_2_**→**^2^A_1_, respectively [19]. Hence, cobalt-doped ZnO is useful for photocatalysis under incandescent light or fluorescent light where little UV light is present.

However, in terms of sustainability, the use of natural sunlight is preferred in photocatalytic applications. Interestingly, under UV light and natural/simulated sunlight, cobalt-doping decreases the photocatalytic activity of ZnO (Table 1). The reduced photocatalytic activity under UV light can be attributed to intra-bandgap impurity levels created by Co^2+^, contributing to enhancing charge-recombination. In the research field of photoluminescence, cobalt is known as the so-called “killer element” to suppress the near-band-edge emission band of ZnO [30]. This suppression occurs due to the partially occupied intra-bandgap impurity levels acting as charge-recombination centers for photo-induced excitons [31]. In a similar manner, the electrons and holes that are excited by UV-light recombine at the cobalt-impurity levels. When this recombination effect is greater than the visible-light excitation of charges, photocatalytic activity is reduced under natural/simulated sunlight.

In contrast to cobalt doping, the influence of manganese doping on the photocatalytic activity of ZnO nanopowders is largely inconclusive. There are many conflicting results reported on the effects of manganese doping. Table 2 lists some recent reports about the effects of manganese-doping on the photocatalytic activity of ZnO nanopowders. Crystal-doped Mn^2+^ in ZnO creates a visible-light absorption band at ~450 nm that is associated with the ^6^A_1_**→**^4^T_1_ electron transition in the tetrahedrally coordinated Mn^2+^ [32]. This absorption band is normally considered as the reason for enhanced photocatalytic activity under visible light. However, as presented in Table 2, under the illumination of UV light and natural/simulated sunlight, the reported changes in photocatalytic activity are not in agreement with each other. The discrepancy appears to be unrelated with the synthesis technique used and doping levels (Table 2).

Similar contradictory results have been reported for other dopant elements including copper [33,34], cerium [35,36] and indium [37,38]. These controversial results should be rectified to develop efficient photocatalysts with desired properties. For this, it is critical to understand the causes of the conflicting results. One of the causes is the lack of knowledge about the locations of dopant ions. The following sections demonstrate how easily one can draw wrong conclusions from the results of widely-used analytical techniques and how this situation can be overcome by combining a group of analytical techniques. We will also discuss that, even when conflicting results are not present, such as in the case of cobalt doping (Table 1), one cannot assume successful bulk crystal doping.

## 3. Common Interpretation: XRD and UV-Vis Absorption Studies

This section reviews the frequently used argument on the results of XRD and UV-Vis absorption measurement studies to interpret the effects of dopants on the photocatalysis of semiconductor nanoparticles. This will help highlight the risks in relying on only these two techniques for the interpretation of the locations of added impurity ions, discussed in the later sections.

**Figure 1:**
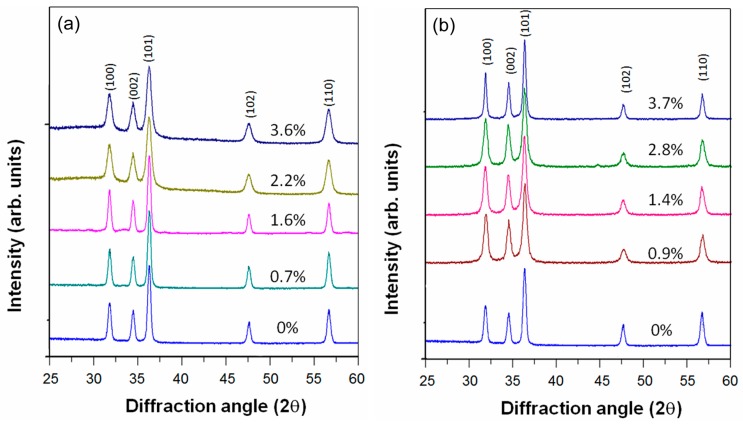
X-ray diffraction (XRD) patterns of (**a**) cobalt-doped and (**b**) manganese-doped ZnO nanopowders.

Figure 1 shows XRD patterns of cobalt- and manganese-ZnO nanopowders [14,15]. Cobalt-doped and manganese-doped ZnO nanopowders were synthesized using a sol-gel co-precipitation method. The details of the synthesis conditions and characterization procedures are described in Ref. [14] and Figures S2–S7. In all the presented data, the true impurity levels determined by energy-dispersive X-ray spectroscopy were used, instead of the atomic ratio of impurity ions in the starting materials (Figure S1). The XRD patterns showed only a single phase wurtzite structure corresponding to ZnO. 

**Figure 2:**
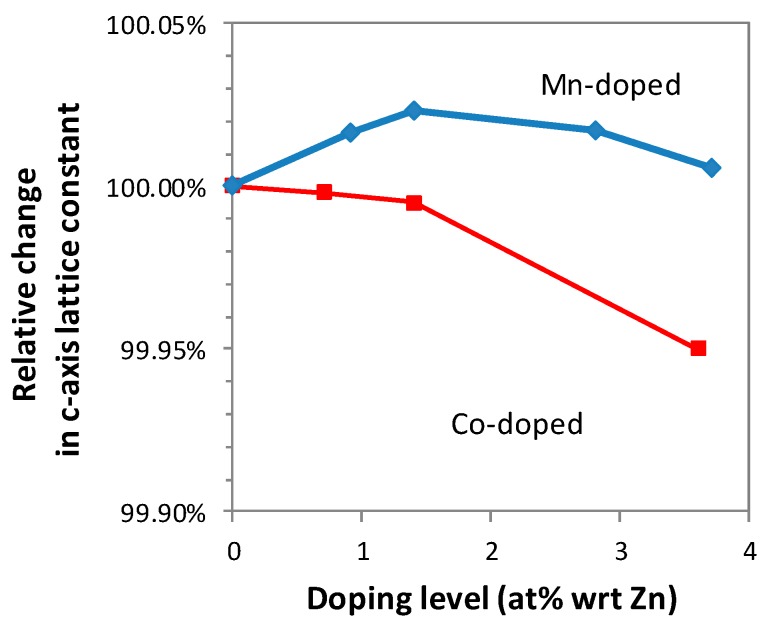
Relative change in c-axis lattice constant obtained from profile fitting of the XRD patterns, for cobalt-doped and manganese-doped ZnO nanopowders.

Figure 2 shows the relative change in c-axis lattice constant as a function of doping level, estimated using the Rietveld analysis. The change in the lattice parameter suggests that lattice expansion and shrinkage were induced by crystal-doping of Co^2+^ (0.72 Å) and Mn^2+^ (0.80 Å) in the Zn^2+^ (0.74 Å) sites in ZnO, due to the slight difference in their ionic radii. The change in lattice parameters is one of the most commonly used pieces of data as evidence of crystal doping. Figure 2 indicates that Mn^2+^ was bulk crystal doped up to ~1.5%, whereas the bulk crystal doping of Co^2+^ was successful up to ~3.6%.

**Figure 3:**
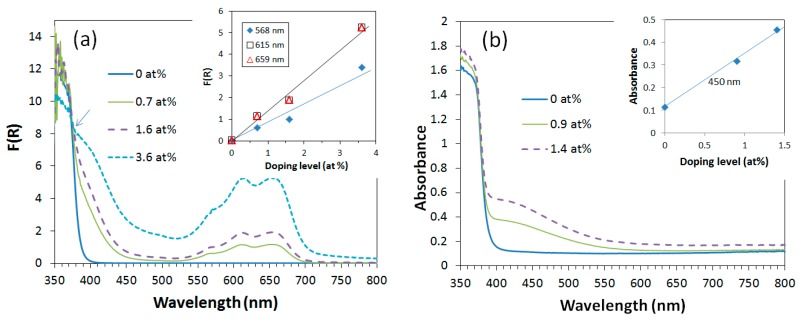
(**a**) Kubelka–Munk function of cobalt-doped ZnO nanopowders; the inset shows the peak height at 568, 615 and 659 nm as a function of doping level. Reproduced with permission from [16]; published by Springer, 2013. (**b**) Absorption spectra of manganese-doped ZnO nanopowders; the inset show the absorbance at 450 nm as a function of doping level.

Figure 3 shows the optical absorption spectra of cobalt-doped [16] and manganese-doped [17] ZnO nanopowders. In Figure 3a, visible-light absorption bands at 659, 615 and 568 nm that are associated with ^4^A_2_**→**^2^E, ^4^A_2_**→**4T1, and ^4^A_2_**→**^2^A_1_ d-d* electron transitions are clearly seen. These bands are associated with tetrahedrally coordinated Co^2+^ [19]. The thermodynamically most stable structure of CoO at room temperature under ambient pressure is a rock salt structure where the cobalt ion is octahedrally coordinated. Hence, this data can be interpreted as evidence that cobalt ions took Zn^2+^ tetrahedral coordination sites in ZnO while retaining the valence number of 2+, instead of forming CoO. Similarly, in Figure 3b, a new absorption band at ~450 nm appeared upon doping of manganese. This band can be assigned to the ^6^A_1_**→**^4^T_1_ electron transition in the tetrahedrally coordinated Mn^2+^ and thus can be taken as evidence for manganese ions having occupied Zn^2+^ tetrahedral coordination sites in ZnO while retaining the valence number of 2+. The intensity of visible-light absorption bands increases proportionally with the doping level, which gives some confidence to this interpretation of the data. 

**Figure 4:**
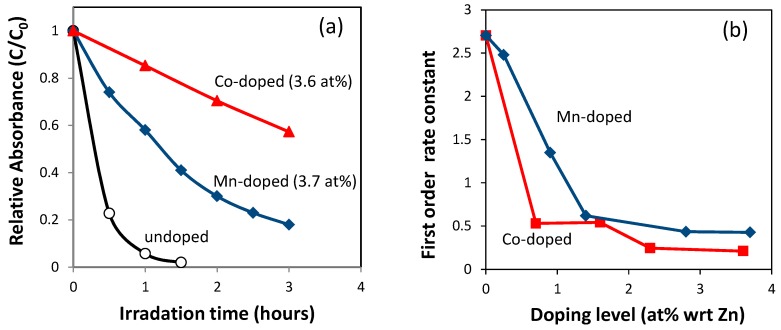
Photocatalytic decomposition of Rhodamine-B by cobalt-doped and manganese-added ZnO nanopowders synthesized using a sol-gel coprecipitation method. (**a**) Relative change in the intensity of optical absorption peak of Rhodamine-B at 554 nm, as a function of irradiation time of simulated sunlight, without normalization by specific surface area values; (**b**) first order rate constant as a function of dopant concentration, normalized with specific surface area. See also Figures S8 and S9.

Figure 4 shows the effects of doping on the photocatalytic degradation of Rhodamine-B under simulated sunlight [14]. The data indicate that both cobalt-doping and manganese-doping up to ~4 at% reduced the photocatalytic activity of ZnO nanopowders. At the same dopant level, cobalt-doping led to a stronger reduction of photoactivity than manganese doping.

Often, these XRD and optical absorption data are the only data used to infer the effect of doping on photocatalysis. The data in Figure 2, Figure 3 and Figure 4 are commonly interpreted as follows. The doping of cobalt and manganese resulted in bulk crystal doping under the doping level of 4% for cobalt and 2% for manganese. The bulk crystal doping created impurity-related intra-bandgap energy levels that in turn contribute to enhanced photon capture by inducing visible-light absorption. Despite this, the overall photocatalytic activity under simulated sunlight was reduced, possibly because the intra-bandgap impurity levels cause the recombination of photo-excited charges. However, reliance on these data alone can result in faulty conclusions as discussed in the next section.

## 4. Atomic-Scale Studies

### 4.1. X-ray Absorption Spectroscopy

Atomic-resolution measurements are useful to study the location of dopant ions in nanoparticles. An example of such a technique is X-ray absorption spectroscopy (XAS). Extended X-ray absorption fine structure (EXAFS) measurements, one of the XAS techniques, enable the determination of the radial distribution of atoms around the dopant ions [29]. The peak positions and peak areas of the radical distribution function are related to the distance and number of surrounding ions, giving information about the local atomic arrangement around the central ions. Another XAS technique, X-ray absorption near-edge structure (XANES) measurements, gives information about the oxidation number and atomic coordination of added impurity ions [49]. Unlike other methods for oxidation state analysis such as X-ray photoelectron spectroscopy, XANES can also be used to identify the location of the element of interest in the host matrix [50].

**Figure 5:**
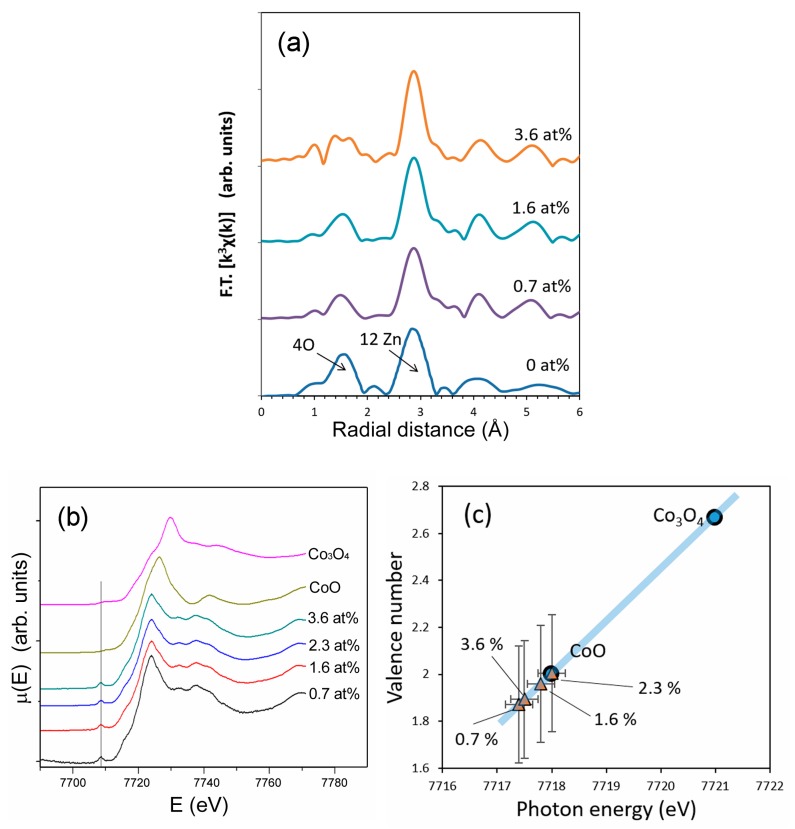
(**a**) Radial distribution function around cobalt ions in undoped ZnO and cobalt-doped ZnO (**b**) X-ray absorption near edge structure (XANES) spectra of cobalt-doped ZnO, CoO and Co_3_O_4_ nanopowders, and (**c**) correlation between the absorption edge energy and oxidation states of cobalt in cobalt-doped ZnO, CoO and Co_3_O_4_ nanopowders. The spectra were taken at Zn K-edge for undoped ZnO and at Co K-edge for cobalt-doped ZnO, CoO and Co_3_O_4_.

Figure 5a shows atomic radial distribution functions around the central zinc ions in ZnO and cobalt ions in doped ZnO, obtained from our previous EXAFS study. As shown in Figure 5a, the local atomic arrangement around cobalt was nearly identical to that around zinc ions. Figure 5b shows XANES spectra of cobalt-doped ZnO and reference samples, CoO and Co_3_O_4_, taken at the cobalt-K-edge. The XANES spectra of cobalt-doped ZnO closely resemble that of CoO with Co^2+^, rather than Co_3_O_4_ with a mixed oxidation state of Co^2+^ and Co^3+^. In general, the oxidation number of ions have a linear correlation with the absorption-edge energy determined using the half-height method [49]. The relation in Figure 5c indicates that the oxidation state of doped cobalt was close to 2+. The vertical error bars in Figure 5c are taken from Ref. [49]. A pre-edge peak around 7710 eV is present in the XANES spectra of cobalt-doped ZnO and the pre-edge peak is associated with the hybridization between p and d states, which is only possible if the location of cobalt does not have an inversion center of symmetry, as in a tetrahedral configuration [50].

The EXAFS and XANES data indicate that a majority of cobalt in the doped ZnO has the oxidation state of 2+ and replaced zinc in the ZnO lattice. The result is commonly used as strong evidence of bulk crystal doping. However, one of the polymorphs of CoO has a wurtzite crystal structure with lattice parameters of a = 0.321 and c = 0.524 nm [51]. This crystal structure is identical to the wurtzite structure of ZnO (a = 0.325 nm, c = 0.520 nm) [52]. Although the thermodynamically most stable crystal structure of CoO is not a wurtzite structure but a rock salt structure [51], nanoparticles may have thermodynamically metastable crystal phases due to large surface areas [53]. Thus, the EXAFS result alone cannot exclude the possibility that CoO with a wurtzite structure precipitated within or on the surface of ZnO nanoparticles. Wurtzite CoO has tetrahedrally coordinated Co^2+^, so that the visible-light absorption bands shown in Figure 3a may be derived from not only the crystal-doped Co^2+^ in ZnO but also the wurtzite CoO phase if it existed. Nonetheless, the peak shift in the XRD patterns (Figure 2) indicates that some, if not all, cobalt ions were crystal doped into ZnO.

**Figure 6:**
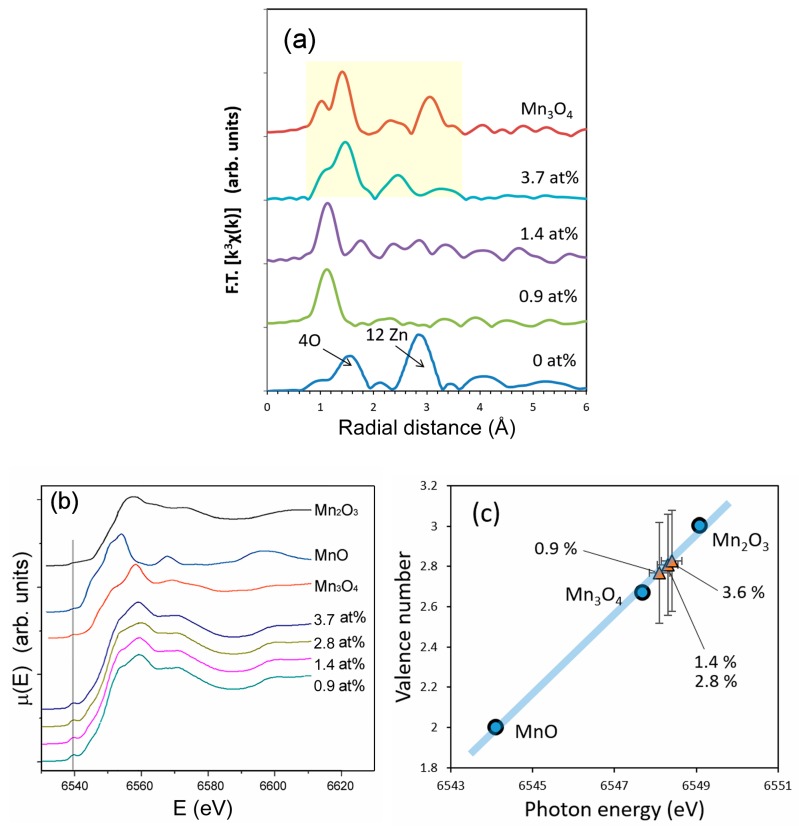
(**a**) Radial distribution function around manganese ions in undoped ZnO and manganese-doped ZnO; (**b**) X-ray absorption near edge structure (XANES) spectra of manganese-doped ZnO, MnO, Mn_2_O_3_ and Mn_3_O_4_ nanopowders, and (**c**) correlation between the absorption edge energy and oxidation states of manganese in manganese-doped ZnO, MnO, Mn_2_O_3_ and Mn_3_O_4_ powders. The spectra were taken at Zn K-edge for undoped ZnO and at Mn K-edge for Mn-doped ZnO, MnO, Mn_2_O_3_ and Mn_3_O_4_.

In contrast to cobalt, the local atomic arrangement around manganese is different from the atomic arrangement around zinc (Figure 6a). When the dopant level is lower than 1.4 at%, the local atomic arrangement around manganese lacks long-range order, suggesting an amorphous-like local structure instead of bulk crystal doping. This result appears to contradict the XRD data that indicated bulk crystal doping (Figure 1). However, the EXAFS results may suggest the possibility that manganese takes more than two locations including zinc sites (bulk crystal doping) and in surface precipitates (surface modification). When the dopant level is increased to 3.4 at%, the local atomic arrangement around manganese in ZnO resembles that in Mn_3_O_4_. This clearly indicates that the majority of manganese is not bulk crystal doped, in good agreement with the XRD data in Figure 1.

The XANES spectra of manganese-doped ZnO resemble those of Mn_3_O_4_ rather than MnO or Mn_2_O_3_. The correlation between the oxidation states and absorption-edge energy determined by the half-height method (Figure 6c) also indicates that the oxidation state of manganese is close to that in Mn_3_O_4_. The presence of a pre-edge peak at ~6540 eV (Figure 6b) indicates that the location of manganese does not have an inversion center of symmetry. However, it does not mean that manganese ions are bulk crystal doped into a tetragonal configuration. Manganese in Mn_3_O_4_ takes a tetragonal configuration, which creates a pre-edge peak at ~6540 eV in the XANES spectra as shown in Figure 6c.

The XAS results of ZnO nanoparticles with manganese impurities are a clear indication that a majority of manganese did not replace zinc in the ZnO lattice. The data suggest that the majority of the manganese compounds precipitated within or on the surface of ZnO nanoparticles. The precipitates have a rather random short range atomic order but the atomic configuration has a certain degree of resemblance to that of Mn_3_O_4._ It is interesting to note that the XRD study was not able to detect either type of precipitates (bulk or surface) as a phase separate from ZnO, possibly due to their small quantity and near-amorphous nature. It should also be noted that the XRD data (Figure 2) and optical absorption data (Figure 3b) suggest that some manganese ions were bulk crystal doped into ZnO, however the degree of bulk crystal doping was not high enough to be shown in the EXAFS data.

### 4.2. High Resolution Scanning Transmission Electron Microscopy Elemental Mapping

High resolution scanning transmission electron microscopy (HR-STEM) is another important technique that provides vital clues for the location of impurity ions [54]. HR-STEM facilities dedicated to capturing elemental maps at or near atomic-resolution, such as the FEI Titan G2 80-200 TEM/STEM equipped with ChemiSTEM Technology, can visualize the distribution of impurity elements within nanoparticles. As demonstrated above, EXAFS measurements provide vital information about the location of dopant elements. However, EXAFS measurements alone do not make it clear where the precipitated/segregated Mn_3_O_4_ is located among the ZnO nanoparticles. HR-STEM elemental mapping can provide this vital information. The visualization of the distribution of impurity ions at such a small scale cannot be achieved by other techniques.

**Figure 7:**
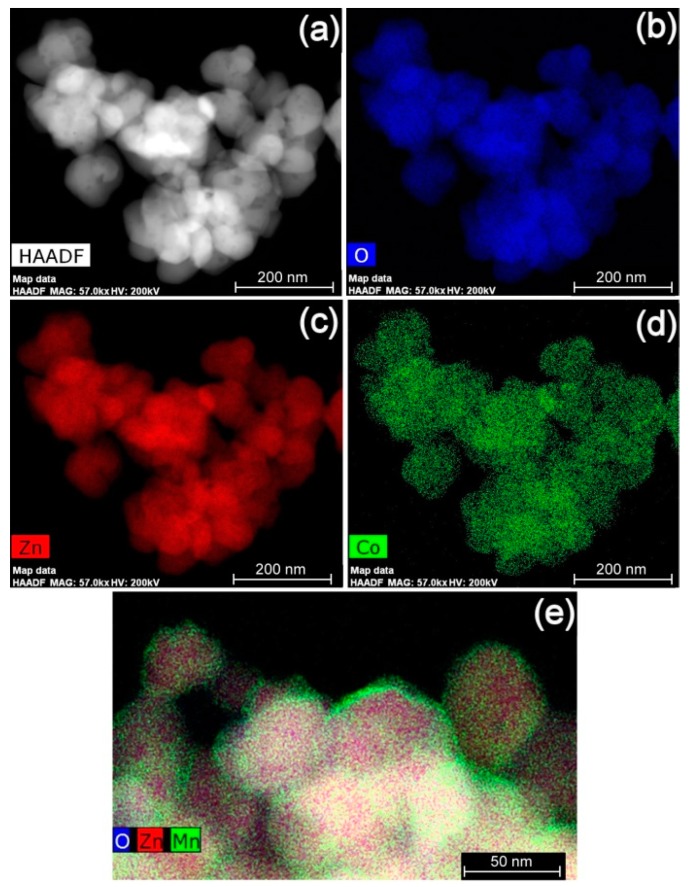
High resolution scanning transmission electron microscopy (HR-STEM) elemental mapping images of 3.6 at% cobalt-doped ZnO nanopowders, synthesized using a sol-gel coprecipitation method: (**a**) high-angle annular dark-field (HAADF) image; (**b**) oxygen map; (**c**) zinc map; (**d**) cobalt map; and (**e**) another area of combined map.

Figure 7 shows elemental mapping images of 3.6 at% cobalt-doped ZnO nanopowders and 3.7 at% manganese-doped ZnO nanopowders. A high-angle annular dark field STEM image (HAADF, representing a Z-contrast image) is also shown in Figure 7a. As shown in Figure 7b–d, all elements are evenly distributed within the particles. All particles contain zinc and no separate particle with only cobalt and oxygen was evident. In some parts of the samples, cobalt appeared to be concentrated near particle surface (Figure 7e). However, this was not a common occurrence as can be seen in Figure 7d. The results strongly indicate that the majority of cobalt is bulk crystal doped into ZnO instead of precipitated out as the wurtzite CoO phase.

**Figure 8:**
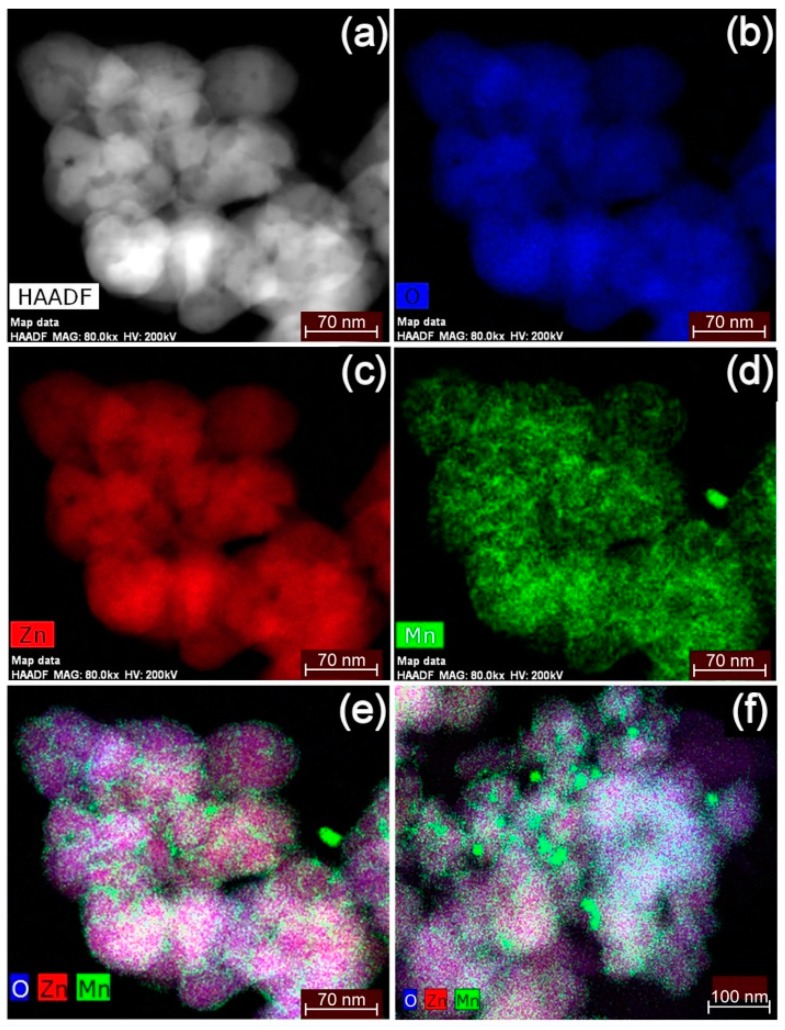
High resolution scanning transmission electron microscopy (HR-STEM) elemental mapping images of 3.7 at% manganese-doped ZnO nanopowders, synthesized using a sol-gel co-precipitation method: (**a**) high-angle annular dark-field (HAADF) image; (**b**) oxygen map; (**c**) zinc map; (**d**) manganese map; (**e**) the combined map for the same area; and (**f**) combined map for another area.

On the other hand, HR-STEM elemental mapping images of manganese-doped ZnO (Figure 8) showed a different scenario. Figure 8d depicts an uneven manganese distribution in doped ZnO. The non-uniform concentration of manganese is clearly visible when the elemental mapping images for zinc, manganese and oxygen are combined (Figure 8e), where manganese appears to be severely segregated from ZnO. In another part of the sample, nanoparticles of manganese oxide with sizes of ~20 nm were evident, alongside ZnO nanoparticles with a relatively homogeneous manganese distribution (Figure 8f). The result suggests that much of the manganese ions are not crystal doped. The precipitated/segregated Mn_3_O_4_ was not detected by an XRD study as discussed earlier. This is because (i) the quantity of precipitated/segregated Mn_3_O_4_ was very low compared to that of ZnO, (ii) the crystallinity of precipitated/segregated Mn_3_O_4_ was poor as shown in the XAS data in Figure 5b, and (iii) the size of Mn_3_O_4_ was very small, which caused further broadening of XRD peaks associated with Mn_3_O_4_.

The results clearly demonstrate the importance of high resolution elemental mapping to determine the location of supposed-to-be doped elements, for which XRD and optical absorbance measurements do not provide sufficient evidence. XAS analysis alone was not sufficient to determine the location of the dopant element, but a combination of XAS and HR-STEM elemental mapping was necessary to confirm the high level of bulk crystal doping in ZnO nanoparticles.

## 5. Cause of Conflicting Results in the Past Studies

### 5.1. Cause of Conflicting Results in the Past Studies

The results of the many analytical techniques presented in this paper show that the added impurities can take more than one location in the host material, resulting in both bulk modification and surface modification. This was revealed only by carefully combining the results from several different characterization techniques. It is not sufficient to use only XRD phase analysis, the shift in the position of XRD peaks and the appearance of new optical absorption bands in the absorption spectra. Even advanced techniques such as EXAFS, XANES and HR-STEM still require holistic analyses coupled with other characterization techniques. In particular, the degree of bulk doping and surface modification, which has a large influence on the photocatalytic activities, can be assessed by using more than one technique. However, published articles often lack in-depth analysis of the location of doped ions, which provides insufficient evidence to discuss the doping effects on the photocatalysis of ZnO.

In this paper, combination of the results from several different characterization techniques enabled us to clarify that, in the example materials used in this paper, cobalt-doped and manganese-added ZnO-nanoparticles made using a sol-gel method, a majority of cobalt was bulk crystal doped, but the majority of manganese decorated the nanoparticle surface as amorphous-like Mn_3_O_4_. This allows one to make a more accurate assessment of the cause of reduced photocatalytic activities in these samples.

The consistency in past reports on the effect of cobalt-doping on the photocatalysis of ZnO (Table 1) can be attributed to the fact that cobalt is readily crystal-doped into ZnO and thus does not produce a large variety of bulk-to-surface modification ratios.

On the other hand, the addition of manganese resulted in a large degree of surface modification with a small degree of bulk modification, along with the formation of a small quantity of segregated Mn_3_O_4_ nanoparticles (Figure 8). It is speculated that the ratios between these three locations/states of manganese ions can change significantly depending on the synthesis techniques used. The oxidation state of the impurity ions may also be altered using different synthesis techniques. The locations and valence states of doped ions can significantly influence the photocatalytic activity of host ZnO. For example, bulk crystal doping of Mn^2+^ may increase the photoactivity in the visible light region, while bulk crystal doping of Mn^3+^ may decrease the photoactivity by acting as strong recombination sites for photo-excited charges. The precipitation of a secondary phase on the particle surface can act in various ways; if the precipitate is a semiconductor where the band edge position favors separation of photo-excited charges, the photoactivity may be enhanced. On the other hand, if the precipitate has the ability to block light, to scavenge free radicals, or to recombine photo-excited charges, the photoactivity may be reduced. As such, the inconsistency in the past reports about the effect of manganese-doping on the photocatalysis of ZnO (Table 2) is attributable to the difference in the locations and oxidation states of manganese in different reports.

**Figure 9:**
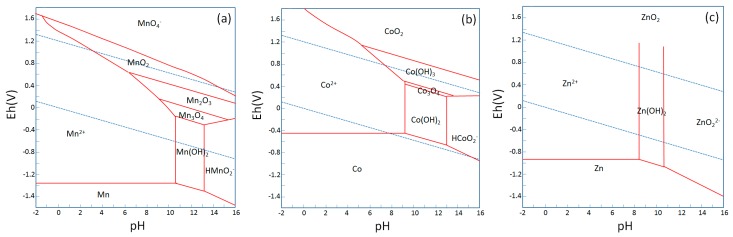
Pourbaix diagram of (**a**) manganese, (**b**) cobalt and (**c**) zinc at 25 °C, 10^5^ Pa, and solution concentration of 10^−6^.

The variation in the location and valence states of manganese ions can occur even using the same synthesis technique. For aqueous-based methods such as sol-gel co-precipitation and hydrothermal synthesis, Pourbaix diagrams give some insight into the way the variation in the location and valence states of doped ions can occur. As can be seen in the Pourbaix diagram of manganese (Figure 9a), manganese ions can precipitate into different valence numbers, depending on the redox environment in water. The diagram suggests that, unless a reducing or oxidizing agent is used, manganese will precipitate into a valence state between +2 and +4, during the sol-gel synthesis of Mn-added ZnO. Figure 9a,c also suggest that, while raising the pH of a Zn^2+^ and Mn^2+^ mixed solution, ZnO may precipitate out at a lower pH than Mn_3_O_4_. If this occurs, Mn_3_O_4_ will likely precipitate on the surface of ZnO as ZnO will act as a nucleation site for Mn_3_O_4_. Thus, the successful bulk crystal doping of Mn^2+^ into ZnO may depend on the speed in changing pH during the co-precipitation process. Figure 9b shows that the onset pH to precipitate CoO from a Co^2+^ solution is similar to that of ZnO and that the valence number of cobalt is likely to stay as 2+ during the sol-gel synthesis of Co-doped ZnO, leading to more successful bulk crystal doping of cobalt in ZnO than manganese. However, the slightly higher onset-pH of cobalt than zinc may cause the concentration of cobalt near the surface of ZnO as shown in Figure 7e. Other conditions such as the influence of the types of anions should also be investigated.

### 5.2. Interpretation of Reduced Photocatalysis Caused by the Addition of Manganese

Interestingly, even when attempted doping of manganese did not result in bulk crystal doping, the change induced in the photocatalytic activity was similar to that of cobalt-doping where the majority of cobalt was bulk crystal-doped (Figure 4). The reduction of photoactivity in manganese-added ZnO cannot be attributed to the charge recombination of photo-excited electrons and holes by the creation of intra-bandgap energy levels. The reason for the reduction of photoactivity by the addition of manganese ions should be sought elsewhere. Here, knowledge about the location of manganese becomes important.

The combination of several different characterization techniques revealed that addition of manganese ions resulted in Mn_3_O_4_ grafted on the surface of the ZnO nanoparticles. Mn_3_O_4_ has photocatalytic properties [55,56] but only at a pH below 4 [57]. In the present study, Rhodamine-B, an acidic dye, was used as a model organic pollutant to examine the photocatalytic activity of manganese-added ZnO. Rhodamine-B and other colored dyes are often used as a model organic pollutant, due partially to the common applications of photocatalysts in purifying industrial effluent that contains colored organic pollutants. However, as Ohtani pointed out [11,12], colored dyes are not well suited to test visible light photocatalytic activities because the dyes can absorb visible light. In our study, the Rhodamine-B solution was highly diluted (0.0096 g/L) to minimize its visible-light absorption and thus had a pH of 6.5. At this near-neutral pH, one cannot expect photocatalytic activity from Mn_3_O_4_ alone [57].

**Figure 10:**
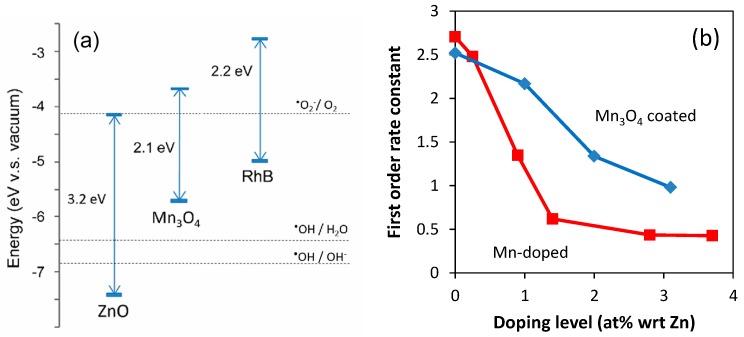
(**a**) Valence and conduction band edge positions of ZnO [63], Mn_3_O_4_ [58], and the positions of highest occupied molecular orbital (HOMO) and lowest unoccupied molecular orbital (LUMO) of Rhodamine-B [64]; (**b**) photocatalytic decomposition of Rhodamine-B by ZnO nanopowders coated with Mn_3_O_4_ and doped with Mn [65]. First order rate constants as a function of Mn concentration with respect to Zn, normalized with specific surface area, are shown.

The Mn_3_O_4_ on the surface of the ZnO nanoparticles may form a heterojunction with ZnO. In order to understand the nature of the heterojunction, the values of the flat-potential (valence band edge position) and bandgap energy of Mn_3_O_4_ are required. There are many different values reported in the past, but recent findings suggest that ZnO and Mn_3_O_4_ can form a Z-scheme to promote the separation of photoinduced charges as shown in Figure 10a [58,59,60,61]. In addition, the positions of highest occupied molecular orbital (HOMO) and lowest unoccupied molecular orbital (LUMO) of Rhodamine-B in Figure 10a suggest that Rhodamine-B may act as a photo-sensitizer to further contribute to charge separation. Higher charge separation efficiency should improve photoactivity. However, our study showed that addition of manganese to ZnO resulted in reduced photoactivity.

The weakening of the photoactivity by the addition of manganese can be attributed to the following:

(1) The surface of ZnO is partially covered with Mn_3_O_4_, which reduces the adsorption of dye molecules on the ZnO surface and in turn reduces the rate of dye degradation by the photocatalysis of ZnO.
(2) The small quantity of segregated Mn_3_O_4_ nanoparticles and Mn_3_O_4_ surface precipitates blocks light coming into ZnO, leading to the reduction of ZnO photocatalysis.
(3) Due to the mixed oxidation state, Mn_3_O_4_ can act as a reducing agent. It is reported that, around neutral pH, Mn_3_O_4_ acts as an anti-oxidant or a free-radical scavenger [62]. The Mn_3_O_4_ precipitates shown in Figure 8 will reduce the number of free radicals that are generated by the photocatalysts of ZnO, leading to the overall reduction of photocatalysis.
(4) A small amount of Mn may be bulk-crystal-doped into ZnO, creating chemical defects that have valence numbers different from that of Zn ions. Such defects may act as charge recombination sites to reduce the number of photo-generated charges.

When undoped ZnO is intentionally coated with Mn_3_O_4_ precipitates, the photoactivity was reduced (Figure 10b, Figures S9 and S16), indicating the occurrence of the above effects (1–3). The reduction of photoactivity was stronger in Mn-added ZnO than Mn_3_O_4_-coated ZnO (Figure 10b)_,_ possibly due to the occurrence of effect (4) in addition to (1–3).

## 6. Other Analytical Techniques

Despite the significant importance of XAS and HR-STEM, these techniques are not widely used. This is because they require significant capital investment such as synchrotron facilities and dedicated TEM laboratories. However, there are other analytical techniques that can be deployed more readily to study the location of impurity ions in nanoparticles.

One such example is X-ray photoelectron spectroscopy (XPS). XPS can characterize the chemical states of elements in the top few nm of the surface. As such, XPS is an important tool to study whether a particular element is either on the surface or in the bulk. For example, Szczuko et al. determined the surface concentration of impurity elements in SnO_2_ fine powder by XPS. [66]. Srinet et al. were able to show that cobalt in sol-gel derived Zn_1-__x_Co_x_O had a divalent state surrounded by oxygen tetrahedra, by studying the binding energy separation between Co2p3/2 and Co 2p1/2 levels [67]. Using the same analytical approach, our XPS results also indicated that cobalt in ZnO had a divalent state surrounded by oxygen tetrahedra (Figure S4) [16]. However, the minimum analysis area of XPS is normally larger than 10 micrometers. Although the use of a synchrotron can reduce the minimum spatial resolution of XPS down to ~200 nm, it is still too large to characterize nanoscopically non-uniform surface modifications such as the ones shown in Figure 7 and Figure 8 and it would not show whether the manganese oxide is on the surface of the ZnO or in a separate phase.

Another example is zeta potential measurement. Zeta potential is sensitive to surface chemistry, so that it helps determine whether the dopant ions are located within ZnO particles, e.g., on grain boundaries or as interstitial defects, or outside of ZnO particles, e.g., on the surface or as separate particles.

**Figure 11:**
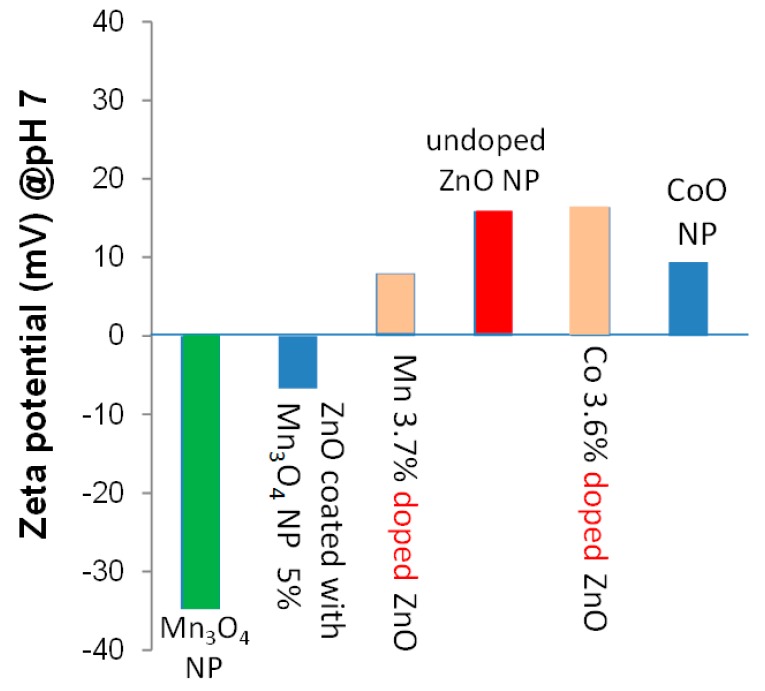
Zeta potential of nanopowders (NPs) at pH 7.

Figure 11 compares the zeta potential of undoped ZnO, cobalt- or manganese- added ZnO, Mn_3_O_4_ and CoO nanoparticles (Figures S10 and S11), and undoped ZnO that is intentionally coated with Mn_3_O_4_ precipitates (Figures S12–S15) [14]. At pH 7, the zeta potentials of ZnO and CoO nanoparticles are approximately +18 mV and +10 mV. Thus, if CoO is precipitated out on the surface of cobalt-doped ZnO, the zeta potential of the doped ZnO is expected to decrease. However, the zeta potential of cobalt-doped ZnO was nearly the same as undoped ZnO. This implies that most of the cobalt ions are located within ZnO nanoparticles.

Mn_3_O_4_ nanoparticles have a large negative zeta potential of approximately −35 mV at pH 7. Because of this, when undoped ZnO is intentionally coated with Mn_3_O_4_ precipitates, the zeta potential of the sample was reduced from +18 mV to −0.7 mV. As such, if Mn_3_O_4_ is segregated from ZnO to be on the surface of ZnO particles, the zeta potential of doped ZnO should decrease from +18 eV. In fact, when 3.7 at% manganese was added, the reduction in zeta potential, from +18 eV to +9 eV, was observed. The results are in good agreement with the results of XAS (Figure 5 and Figure 6) and HR-STEM (Figure 7 and Figure 8) studies.

By combining the zeta potential study, HR-STEM images and other analytical techniques, one may be able to develop a new approach to quantitatively analyze the ratio between bulk modification and surface modification caused by the addition of impurity ions, in the future. For this, the detection of secondary phases separated from the host, such as Mn_3_O_4_ nanoparticles shown in Figure 8f, would become important.

There are other techniques to obtain evidence of possible bulk modification by crystal doping. For example, Raman spectroscopy can detect disruption of crystalline symmetry by dopants or impurities in a crystal, in the form of peak broadening or shifting of peak position [68]. However, the technique is mainly applied to large single crystals and the detection of the location of impurities in nanoparticles with inhomogeneous and partial surface modification is yet to be demonstrated. In particular, the peak broadening caused by the reduction of crystallite size should be carefully considered for data interpretation [69]. Cathodoluminescence is another technique recently proposed [16]. A change in the position and intensity of phonon-coupling emission bands is expected to occur by the distortion of crystal symmetry [19]. These techniques focus on the detection of possible bulk crystal doping by studying lattice distortion, similar to the conventional use of XRD as discussed earlier. Hence, it is still necessary to combine these techniques with other characterization methods that can detect the presence of surface modification, for the overall analysis of the location of impurity/dopant ions.

## 7. Conclusions

Although impurity doping is one of the common approaches to enhance the photoactivity of semiconductor nanomaterials, it is not straightforward to accurately assess the final resting place of impurity ions at the nanoscale. Intended bulk modification often results in surface modification or precipitation of separate particulate phases. This situation gives rise to inaccurate interpretation of the doping effects and impedes the development of new photocatalytic nanomaterials. This paper demonstrates the challenges in obtaining clear evidence for successful bulk crystal doping, by using transition-metal-doped ZnO nanoparticles as an example case. It was shown that most of the commonly used techniques such as XRD focus on the detection of possible bulk crystal doping (bulk modification) and hence heavy reliance on these techniques may result in inaccurate interpretation of doping effects. Even atomic-scale analyses such as XAS may in some cases provide inconclusive results. Thus, it is important to combine other techniques that can detect surface modification, such as HR-STEM.

The differences in synthesis methods also induce variations in the location and valence states of dopant ions in ZnO. However, the variation in the location and valence states of dopant ions can occur even using the same synthesis technique. It is encouraged that each sample be carefully examined to elucidate the doping effect without ambiguity.

What is still lacking today is a method to quantitatively analyze the different dopant states within the same sample, i.e., how much of the dopant element is crystal doped (bulk modification) and how much resulted in other states. It is essential to establish the methodology to quantitatively analyze doping states in nanoscale particles, for the advancement of photocatalytic materials research.

## Figures and Tables

**Table 1:** Effects of cobalt doping on the photocatalysis of ZnO nanopowders.

Doping at%	Excitation Wavelength Range	Change in Photocatalysis	Synthesis Methods	Reference
0–15	Visible light	Enhanced	Solvo-thermal	[20]
0–1.5	Visible light	Enhanced	Solvo-thermal	[21]
0–5	Visible light	Enhanced	Hydro-thermal	[22]
0–10	Visible light	Enhanced	Coprecipitation (sol-gel)	[23]
0–5	UV light	Reduced	Coprecipitation (sol-gel)	[24]
1, 10	UVC (Hg lamp)	Reduced	Coprecipitation (sol-gel)	[25]
0–9	UVA (365 nm)	Reduced	Coprecipitation (sol-gel)	[26]
0–10	UVA (365 nm)	Reduced	Coprecipitation (sol-gel)	[23]
0–10	Natural sunlight	Identical	Coprecipitation (sol-gel)	[27]
0–5	Simulated sunlight	Reduced	Coprecipitation (sol-gel)	[15]
0–5	Hg-Xe lamp	Reduced	Mechanochemical	[28]
0–5	Simulated sunlight	Reduced	Mechanochemical	[29]

**Table 2:** Effects of manganese doping on the photocatalysis of ZnO nanoparticles.

Doping at%	Wavelength Range	Change in Photocatalysis	Synthesis Methods	Reference
0–8.3	Visible light	Enhanced	Coprecipitation (sol-gel)	[39]
6.3	Visible light	Enhanced	Hydro-thermal	[40]
0–20	Visible light	Enhanced	Solvothermal	[41]
0–7	UV (Ne lamp)	Enhanced	Solvo-thermal	[42]
1.8	UVA (365 nm)	Enhanced	Coprecipitation (sol-gel)	[43]
0–5	UVC (Hg lamp)	Enhanced	Solvo-thermal	[44]
3.6	UVC (Hg lamp)	Reduced	Hydro-thermal	[45]
1.0	UV	Reduced	Thermal decomposition	[46]
0.25	UVA (365 nm)	Reduced	Coprecipitation (sol-gel)	[47]
6	Natural sunlight	Enhanced	Coprecipitation (sol-gel)	[48]
0–5	Hg-Xe lamp	Enhanced	Mechanochemical	[28]
0–5	Simulated sunlight	Reduced	Mechanochemical	[29]
0–5	Simulated sunlight	Reduced	Coprecipitation (sol-gel)	[14]

## Supplementary Materials

The following are available online at https://www.mdpi.com/2079-4991/9/3/481/s1, Figure S1: Correlation between the real doping levels and theoretical doping levels measured by energy-dispersive X-ray spectroscopy (EDX), Figure S2: Brunauer–Emmett–Teller (BET) specific surface areas of undoped, Co-doped and Mn-added ZnO, Figure S3: Volume-weighted particle size distribution of 1.2 at% Mn-added ZnO nanoparticles measured by the dynamic light scattering method, Figure S4: XPS spectra of 2.2 at% Co-doped ZnO nanoparticles, Figure S5: TEM images of (a) undoped, (b) 3.6 at% Co-doped and (c) 3.7 at% Mn-added ZnO nanoparticles, Figure S6: Estimation of band gap energies of undoped and Co-doped ZnO using the Tauc plot, Figure S7: Estimation of band gap energies of undoped and Mn-added ZnO using the Tauc plot, Figure S8: Relative change in the intensity of the optical absorption peak at 554 nm of Rhodamine-B as a function of irradiation time and estimate reaction speed with Co-doped ZnO, Figure S9: Relative change in the intensity of the optical absorption peak at 554 nm of Rhodamine-B as a function of irradiation time and estimate reaction speed with Mn-added ZnO, Figure S10: XRD patterns of Mn_3_O_4_ particles used for zeta potential measurements, Figure S11: XRD patterns of CoO used for zeta potential measurements, Figure S12: Correlation between the real doping levels and theoretical doping levels of Mn_3_O_4_- coated ZnO tested by EDX, Figure S13: XRD patterns of Mn_3_O_4_- coated ZnO, Figure S14: TEM image of Mn_3_O_4_-coated ZnO, Figure S15: UV-Vis transmittance spectra of Mn_3_O_4_- coated ZnO, Figure S16: Relative change in the intensity of the optical absorption peak at 554 nm of Rhodamine-B as a function of irradiation time and estimate reaction speed with Mn_3_O_4_- coated ZnO.
