# Supplementary material for: Challenges in Determining the Location of Dopants, to Study the Influence of Metal Doping on the Photocatalytic Activities of ZnO Nanopowders
Source: Nanomaterials (Basel). 2019 Mar 25;9(3):481. doi: 10.3390/nano9030481 (PMC6474108; doi:10.3390/nano9030481)
Supplement: Supplementary file 1 [file nanomaterials-09-00481-s001.pdf]

Supplementary information

Challenges in Determining the Location of Dopants, to Study the Influence of Metal Doping on the Photocatalytic Activities of ZnO Nanopowders

Takuya Tsuzuki [1,*], Rongliang He [2], Aaron Dodd [3] and Martin Saunders [3]

[1] Research School of Electric, Energy and Materials Engineering, College of Engineering and Computer Science, Australian National University, Canberra 0200, Australia
[2] Institute for Frontier Materials, Deakin University, Waurn Ponds 3216, Australia; rongliang.he@deakin.edu.au
[3] Centre for Microscopy, Characterization and Analysis, The University of Western Australia, 35 Stirling Highway, Perth 6009, Australia; aaron.dodd@uwa.edu.au (A.D.); martin.saunders@uwa.edu.au (M.S.)
* Correspondence: takuya.tsuzuki@anu.edu.au; Tel.: +61-2-6125-9296

1. Note

The citation numbers in this document correspond to those in the main article.

2. Synthesis conditions

For the present article, analytical results were gathered from our previously published papers [14-17], except newly acquired High Angle Annular Dark Field Scanning Transmission Electron Microscopy (HAADF-STEM) images and elemental mapping images. The details of sample preparation methods are described in Refs [14-17]. The following provides brief descriptions of the sample preparation conditions.

2.1. Doped ZnO

The details of sample preparation methods are described in Refs [14-17]. Briefly, Co-doped and Mn-doped ZnO were synthesized separately by using a sol–gel co-precipitation method to form doped zinc carbonate hydroxide and subsequent heat treatment to decompose doped zinc carbonate hydroxide into doped ZnO. The raw materials used were $Zn(CH_3COO)_2{\cdot}2H_2O$, (Aldrich [99.0%), NaOH pellets (Chem-Supply 97%), Na_2CO_3 (Aldrich [99.0%), $CoCl_2{\cdot}6H_2O$ (Aldrich, 98.0%), and $Mn(CH_3COO)_2$ (Fluka, 98%), all of which were used without further purification. First, a mixed transparent solution-A consisting of Zn^{2+} and dopant ions with a fixed atomic ratio (Dopant:Zn^{2+} = 1:99, 1:49, 1:32.3 and 1:19) was prepared in distilled water. At the same time, NaOH and Na_2CO_3 were also dissolved in distilled water to prepare another solution-B which would be used as a precipitant. Then, the solution-A was added into the solution-B drop-wise with continued magnetic stirring at the speed of 500 rpm. The precipitate was immediately formed during the mixing of the two solutions. After 1.5 h of aging, the slurries were washed with distilled water several times using a centrifuge at the speed of 7,000 rpm (Eppendorf centrifuge 5417R) until the salinity of the supernatant becomes less than 100 ppm. The washed powders were oven-dried under 80°C. Finally, the dried samples were heat-treated at 400°C for 1 h to decompose doped Zn carbonate hydroxide into doped ZnO.

2.2. *CoO particles*

CoO particles for zeta potential measurements were produced by thermally decomposing cobalt acetate under nitrogen-gas flow (100 mL/min) in a tube furnace at 400°C for 2 h. The crystal phase was determined by X-ray diffraction (XRD).

2.3. *Mn_3O_4 particles*

Mn_3O_4 particles for zeta potential measurements were synthesised from $Mn(CH_3COO)_2$, using the same procedure as doped ZnO. The crystal structure was confirmed by XRD.

2.4. *Undoped ZnO coated with Mn_3O_4*

Uncoated pristine ZnO nanopowder was synthesized by a sol-gel co-precipitation method as described above. First, a Mn solution (Solution-A) was prepared in distilled water from $Mn(CH_3COO)_2$ and undoped ZnO particles were dispersed in the solution. Separately, NaOH and Na_2CO_3 were dissolved in distilled water together to prepare a solution-B. Then, the solution-A was added into the solution-B drop-wise while stirring at 500 rpm. The precipitate was immediately formed during the mixing of the two solutions. The Mn-containing precipitate appeared brown. After 1.5 hours of aging, the slurries were washed with distilled water several times using a centrifuge at 7000 rpm (Eppendorf centrifuge 5417R) until the salinity of the supernatant becomes less than 100 ppm. The washed powders were oven dried under 80°C. Finally, the dried samples were heat-treated at 400°C for 1 hour to decompose hydroxide coatings into oxides. The crystal phase of the precipitate was determined to be Mn_3O_4 by XRD, and the quantity of manganese was determined by energy-dispersive X-ray spectroscopy on a Leica LEO S440 scanning electron microscopy.

2.5. *XAS reference samples*

As the reference samples for Extended X-ray absorption fine structure (EXAFS) and X-ray absorption near-edge structure (XANES) measurements, CoO, Co_3O_4, MnO, Mn_2O_3 and Mn_3O_4 were purchased from Sigma Aldrich Australia. The reagents were used without further purification.

3. Analytical measurements

For the present paper, High Angle Annular Dark Field Scanning Transmission Electron Microscopy (HAADF-STEM) images and elemental mapping images were newly obtained. Other results were gathered from our previously published papers. The details of other characterisation techniques are described in Refs [14-17]. Some data, such as XRD patterns, UV-Vis absorption spectra of manganese-added samples, and the X-ray absorption edge energy in XANES spectra, were re-analyzed for this paper.

2.1. STEM: High resolution STEM study was carried out using a FEI Titan G2 80-200 TEM/STEM with ChemiSTEM Technology operating at 200 kV. HAADF-STEM images and elemental mapping were acquired by energy dispersive X-ray spectroscopy using the Super-X detector on the Titan with a probe size ~1 nm and a probe current of ~0.4 nA.

2.2. X-ray diffraction (XRD): The crystal phase of the manganese-added samples was studied using an X-ray diffractometer (Panalytical X'Pert PRO MRD) with Cu K radiation, at an operation voltage and current of 40 kV and 30 mA, respectively. The crystal phase of the cobalt-doped samples was characterized by XRD measurements at the X-ray powder diffraction beamline

(wavelength = 0.82591Å) at the Australian Synchrotron. Lattice parameters were calculated from profile fitting of the XRD patterns from 25 to 60 degrees. Corundum powder and metal titanium powder were used as an internal standard material.

2.3. Optical spectra: A Varian Cary 3E spectrophotometer was used to measure the diffuse reflectance optical absorption spectra of the washed ZnO and doped ZnO powders at a wavelength range of 200 to 800 nm. Bandgap energies were estimated using the Tauc plot methods.

2.4. Doping concentration: The actual doping levels in the powders were characterized using a Varian SPECTRAA-240 atomic absorption spectroscopy instrument in the flame mode. For this, 0.2g of the nanopowder sample was dissolved in 20 ml of 30 wt% HNO_3 solution at 140 ^oC in an autoclave. The solution was further diluted to 50 ml for the measurements.

2.5. Specific surface area: The specific surface area of the particles was analyzed by the Brunauer–Emmett–Teller (BET) gas absorption method using a Micromeritics Tristar 3000 system. All powders were dried at 150 ^oC for 1 hour under a high purity nitrogen gas flow, prior to analysis. The result was used for the analysis of photocatalytic activity.

2.6. X-ray absorption spectroscopy (XAS): XAS experiments were performed at the BL20B beamline (Australian National Beamline Facility) which was located in Photon Factory, Tsukuba, Japan. The samples were diluted with boron nitride to minimize the effects of self absorption and then filled in the 5 × 10 mm^2 cells on the sample holder. The K-edge spectra of Zn, Mn, and Co elements were measured separately in a fluorescence mode. The EXAFS data analysis was carried out using the Ifeffit software package. X-ray absorption edge position was analysed using the half-height method.

2.7. Zeta potential: Zeta potential was measured using a dynamic light scattering (DLS) method with a Malvern Zetasizer-Nano instrument. The as-prepared samples were dispersed in distilled water to 0.01g/L at pH =7, by ultrasonication, without using dispersion agents.

2.8. Energy-dispersive X-ray spectroscopy (EDX): In order to determine the true concentration of impurity ions, EDX was performed, using a Leica 440 SEM at a working voltage of 15 kV. Three different locations on the sample were selected for EDX measurements and the collected results were averaged.

2.9. Transmission electron microscopy (TEM): In addition to HR-STEM study, TEM study was carried out to analyse the morphology of the synthesized particles, using a Philips CM-120 microscope with the beam energy of 120 kV. TEM specimens were prepared by evaporating a drop of the nanoparticle dispersion on a carbon-coated copper grid.

2.10. X-ray photoelectorn spectroscopy (XPS): XPS measurements were performed to study the chemical bonding states of the cobalt ions in the Co-doped ZnO nanoparticles, using a ESCALAB 250 spectrometer (Thermo Fisher Scientific, USA) with a Mg Ka (1253.6 eV) achromatic X-ray source. Survey scans were taken at an analyser-pass energy of 160 eV.

2.11. Particle sizing: Particle size distributions were measured by photo-correlation spectroscopy, using a Malvern Zetasizer Nano. The particles were dispersed in water by ultrasonication prior to the measurements.

2.12. Photocatalytic activity: The photocatalytic activity of the washed powders was measured by monitoring the degradation of Rhodamine-B (RhB) dye in water, in the presence of the powder samples under simulated sunlight irradiated using an ATLAS Suntest equipment. For each measurement, 0.012 g of the powder sample was added into 100 mL of RhB aqueous solution having the concentration of 0.0096 g/L. This small concentration was selected so that RhB will not cause extensive blockage of visible light [11.12]. The temperature of the solution was

regulated to 37°C and the simulated sunlight was irradiated up to 3 h. 3 mL of the suspension was extracted every 30 min and then centrifuged to separate the nanoparticles from the supernatant. UV–Vis absorbance spectra of the supernatant were measured using a Varian Cary 3E spectrophotometer. The intensity of the optical adsorption peak around 554 nm, which is a characteristic absorption band of RhB, was used to monitor the rate of dye degradation. The degradation rate constant was estimated assuing pseudo first order kinetics. The rate constant was normalised with the specific surface area of the samples.

4. Analytical data

4.1. Characteristics of doped ZnO nanoparticles

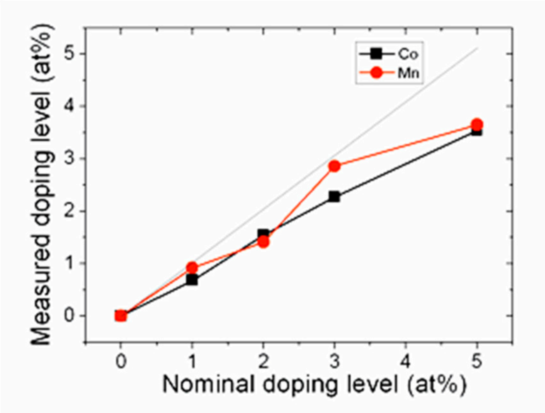

Figure S1. Correlation between the real doping levels and theoretical doping levels measured by EDX [66].

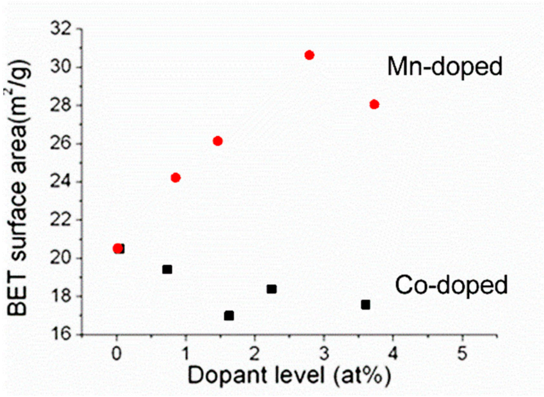

Figure S2. BET specific surface areas of undoped, Co-doped and Mn-added ZnO.

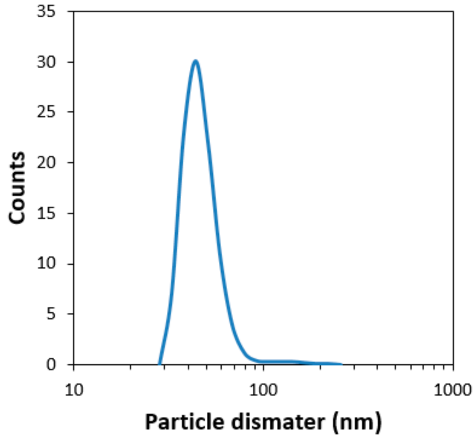

Figure S3. Volume-weighted particle size distribution of 1.2 at% Mn-added ZnO nanoparticles measured by the dynamic light scattering method. The particle size distribution of powders with other doping concentration shows very similar distribution profiles.

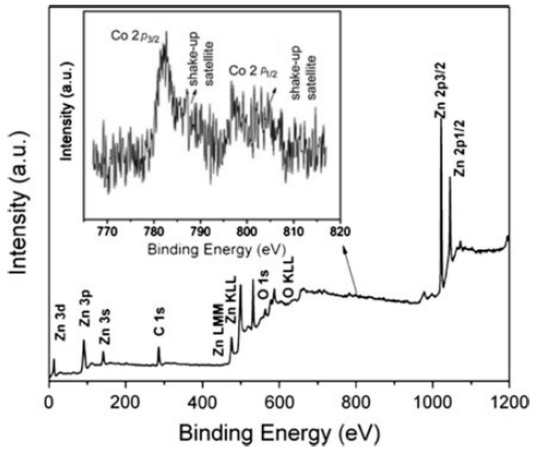

Figure S4. XPS spectra of 2.2 at% Co-doped ZnO nanoparticles. The inset implicates the high resolution XPS spectrum around peaks associated with Co 2p states. The position of the satellite peak at 788.2eV is characteristics of Co^{2+}. Reproduced with permission from [16]; published by Springer, 2013.

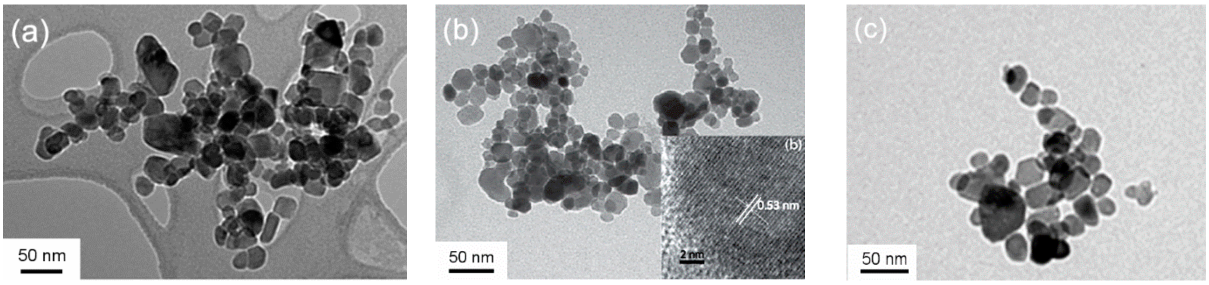

Figure S5. TEM images of (a) undoped, (b) 3.6 at% Co-doped and (c) 3.7 at% Mn-added ZnO nanoparticles. Figure S5(b) is reproduced with permission from [15]; published by Elsevier, 2012. Figure S5(c) is reproduced with permission from [14]; published by Springer, 2012.

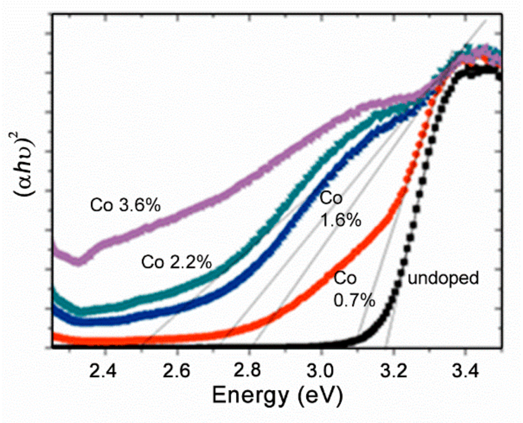

Figure S6. Estimation of band gap energies of undoped and Co-doped ZnO using the Tauc plot [66].

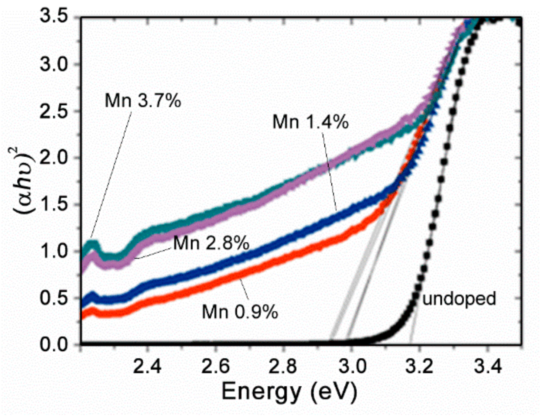

Figure S7. Estimation of band gap energies of undoped and Mn-added ZnO using the Tauc plot [66].

4.2. Rhodamine-B photo-degradation with Co-doped and Mn-added ZnO

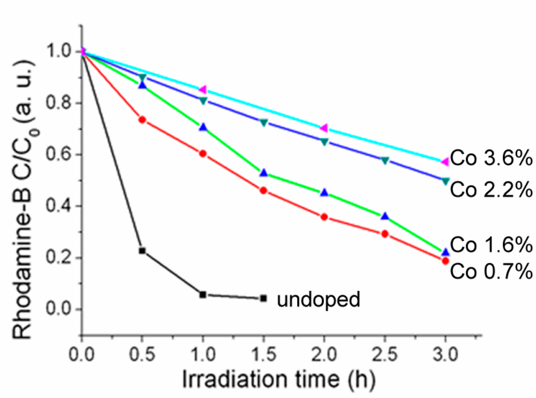

Figure S8. Relative change in the intensity of the optical absorption peak at 554 nm of Rhodamine-B as a function of irradiation time and estimate reaction speed, with Co-doped ZnO [66].

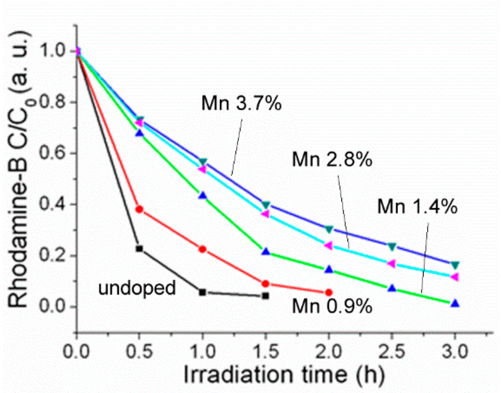

Figure S9. Relative change in the intensity of the optical absorption peak at 554 nm of Rhodamine-B as a function of irradiation time and estimate reaction speed, with Mn-added ZnO [66].

4.3. Characteristics of CoO and Mn_3O_4 particles used for zeta potential measurements

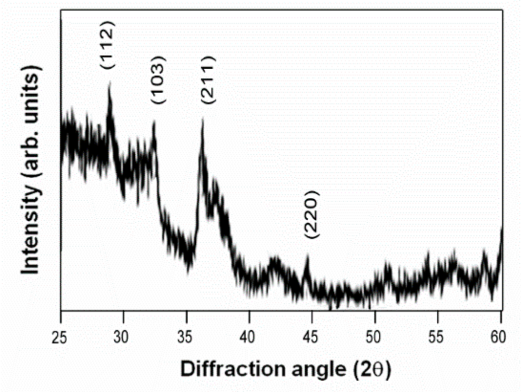

Figure S10. XRD patterns of Mn_3O_4 particles used for zeta potential measurements.

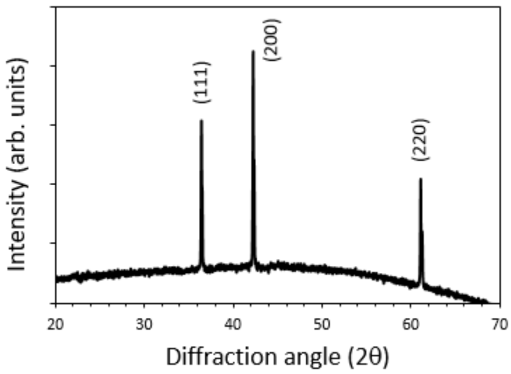

Figure S11. XRD patterns of CoO used for zeta potential measurements.

4.4. Characteristics of Mn-coated ZnO and Rhodamine-B photo-degradation with Mn-coated ZnO

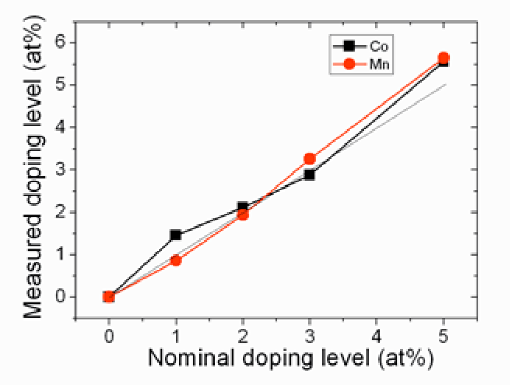

Figure S12. Correlation between the real doping levels and theoretical doping levels of Mn_3O_4-coated ZnO, tested by EDX [66].

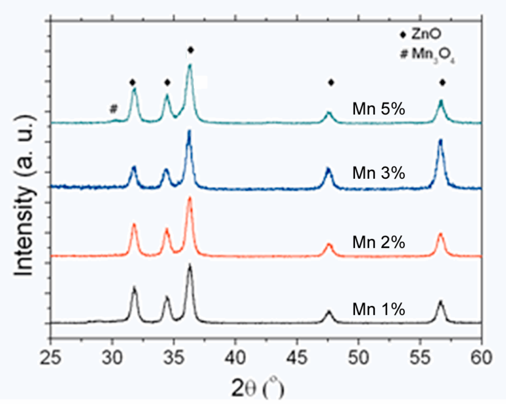

Figure S13. XRD patterns of Mn_3O_4- coated ZnO [66].

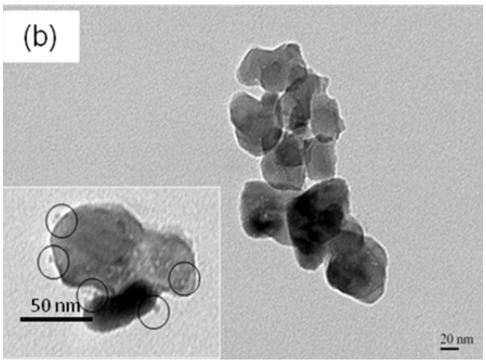

Figure S14. TEM image of Mn_3O_4-coated ZnO. Manganese to zinc atomic ratio is 3:97. Small Mn_3O_4 nanoparticles precipitates are evident on ZnO nanoparticles [66].

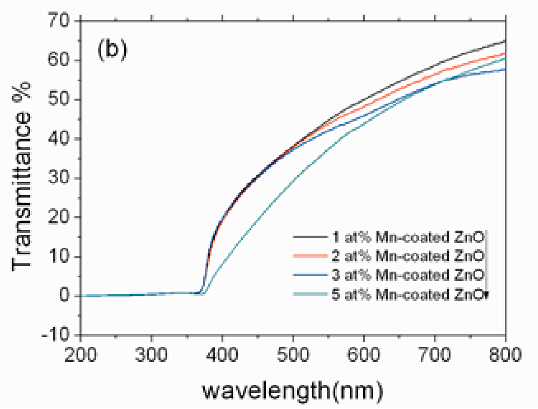

Figure S15. UV-Vis transmittance spectra of Mn_3O_4-coated ZnO [66].

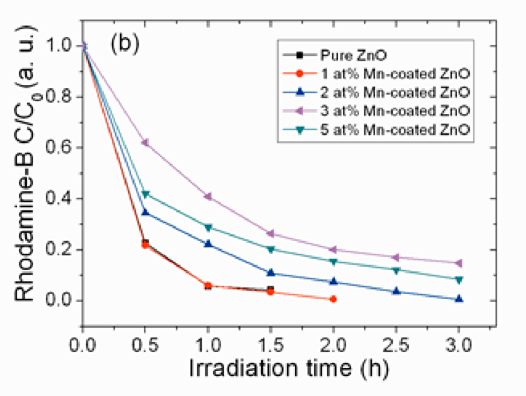

Figure S16. Relative change in the intensity of the optical absorption peak at 554 nm of Rhodamine-B as a function of irradiation time and estimate reaction speed, with Mn_3O_4-coated ZnO [66].
